# Supplementary material for: Molecular Characterization, Recombinant Expression, and Functional Analysis of Carboxypeptidase B in Litopenaeus vannamei
Source: Genes (Basel). 2025 Jan 9;16(1):69. doi: 10.3390/genes16010069 (PMC11764914; doi:10.3390/genes16010069)
Supplement: Supplementary file 1 [file genes-16-00069-s001.zip › Data S1 Lv-CPB sequences.pdf]

Data S1. Gene and protein sequences of Lv-CPB

>Lv-CPB_Gene
AGGCACCTTATATAGGCGGTTGCCAAGGCCGTGTTCTGCACAGACTCGCTCGCCATG
AGGTTCCTGGTCGTTCTCGCCTGCCTGGTGCTGGCGGCGCTCGCTCGCCCCAGCACG
CACGACTACCTGCACGGAGCTCAGGTTCTCCGAGTGAACCCCCAAACAGCTGACCAG
GTTCACTACCTCCAGGGTCTTCTGAAGACTGACCTGTATGACTTCTGGACTGAACCC
CATGGCACTGGTCACCCAGTCGACATCATGGCCCAGGCATTTAGCGTCCCCGTCTTG
AAGAAGACCCTCGAGCAGATTGACCTCGACTTCACCATCCAGGTCAGCGACGTGGCC
CTTCTACTGGCCAAGGACAGGGAGGCCAACCAGAAGGCTCGAGCTGCTTCCGGGAAG
GCCATGGACTGGACCTCGTACCATCGCTATGATGAGATCATGGCCTGGCTGGACGAA
CTGGCGTCCACGCACCCCAGTCTGTGCTCCGTGAAGGAAGTGGGCACAACCTATGAG
GGAAGGACCATGAAAATGCTGACGCTCAACAAGGGAGGAACCGACAAGCCCGGGATC
TTCATCGATGGAGGCATCCACGCCCGCGAGTGGATTTCTCCCGCCACCGTGACCTAC
ATGCTGAACGAGCTGGTCACCAACAGCGACACCTACGACGACATCTTGTCCGCCGTG
AACTTCTACGTTATGCCTTCCATTAACCCCGACGGCTACGAGTACTGCCACACCGAC
GATAGGTTGTGGCGTAAGACTCGCTCTGACAACGGTTCTCCTTTCGGTTGTAAGGGA
GCCGATCCCAACAGGAACTGGGGACACCACTGGAACGAGAACGGCGCCTCAGACAAC
CCCTGCTCGGACATCTACGCCGGTCCCGAAGCCTTCTCAGAGATCGAGATGAAGAAC
GTGCGCAACCAGATCCTCGACCAGACGAACCTGGTGGTGTACCTGACCTTCCACTCC
TACTCCCAGCTGTGGCTCTACCCTTGGGGCTACACTTCAGCTCTCCCCGAGGATTGG
CAGGATTTGGATGACTTGGCCCAATCCGCCGTGAACGCTCTCACAGCTGTGCACGGA
ACCATCTACGACATCGGATCCTCCACCAACGTTTTGTACGCCGCTGCCGGAGGGTCG
GACGACTGGGCCAAGGGCGAAGGCAACGTCAAGTACTCCTACACGGTGGAACTCCGA
GACACGGGCAACTACGGCTTCGTCCTTCCCCCTGACCAGATTATCCCCACCGGAGAG
GAGACCTTCGAAGCCCTCAAGGTCGTCGCGAACTTCGTCAAGGACAACTACCGCACG
CCTTAGAGGGGGTGGTCTCGCGAGAGGCTTGGACCGGACGTCTTGAAAGAAGAACGG
CGAGGGTGTGTGGGTGGGAGGAGGAAATAAATGTGATTTGTGGA

>Lv-CPB_Protein
MRFLVVLACLVLAALARPSTHDYLHGAQVLRVNPQTADQVHYLQGLLKTDLYDFWTE
PHGTGHPVDIMAQAFSVPVLKKTLEQIDLDFTIQVSDVALLLAKDREANQKARAASG
KAMDWTSYHRYDEIMAWLDELASTHPSLCSVKEVGTTYEGRTMKMLTLNKGGTDKPG
IFIDGGIHAREWISPATVTYMLNELVTNSDTYDDILSAVNFYVMPSINPDGYEYCHT
DDRLWRKTRSDNGSPFGCKGADPNRNWGHHWNENGASDNPCSDIYAGPEAFSEIEMK
NVRNQILDQTNLVVYLTFHSYSQLWLYPWGYTSALPEDWQDLDDLAQSAVNALTAVH
GTIYDIGSSTNVLYAAAGGSDDWAKGEGNVKYSYTVELRDTGNYGFVLPPDQIIPTG
EETFEALKVVANFVKDNYRTP

>Lv-CPB_Gene_Optimized
ATGAGGTTTTTGGTTGTATTAGCTTGTCTAGTCCTCGCAGCTCTAGCGCGTCCGTCC
ACCCACGATTATCTTCACGGCGCTCAGGTGTTGCGCGTGAACCCACAGACGGCTGAC

CAAGTGCACTATCTGCAAGGTCTGCTTAAGACCGATCTGTACGACTTTTGGACCGAA
CCGCACGGTACTGGCCACCCAGTCGATATCATGGCGCAAGCGTTCAGCGTTCCGGTA
TTGAAGAAAACGCTGGAGCAGATCGACTTAGACTTCACCATTCAGGTCAGCGATGTT
GCCTTGCTGCTGGCCAAGGATCGCGAGGCAAACCAAAAAGCACGCGCAGCATCCGGT
AAAGCCATGGATTGGACCAGCTACCATCGTTATGACGAAATTATGGCGTGGCTCGAC
GAGCTGGCGAGCACGCACCCGTCACTGTGCAGCGTTAAAGAGGTGGGTACCACTTAC
GAGGGCCGTACCATGAAAATGCTGACCCTGAATAAAGGTGGTACGGACAAACCGGGT
ATATTTATTGACGGCGGGATCCATGCACGCGAATGGATTAGCCCGGCGACCGTTACT
TACATGCTGAATGAATTGGTTACCAACAGCGATACTTATGATGACATCCTGAGCGCT
GTTAACTTCTATGTTATGCCGAGCATTAATCCGGACGGTTATGAATACTGCCACACC
GATGATCGTCTGTGGCGTAAAACCCGTTCTGACAATGGCTCCCCGTTTGGCTGCAAA
GGAGCGGATCCGAATCGTAATTGGGGTCATCATTGGAACGAGAACGGCGCGTCTGAC
AACCCGTGTTCCGACATCTACGCTGGTCCGGAGGCGTTTTCGGAAATTGAGATGAAG
AACGTGCGTAACCAGATCCTGGATCAGACCAACCTGGTTGTGTATCTGACGTTCCAT
AGCTACAGCCAGCTGTGGCTGTACCCATGGGGCTACACCTCTGCTCTGCCGGAAGAT
TGGCAGGATCTGGACGACTTGGCCCAAAGCGCGGTCAACGCGTTAACCGCCGTTCAC
GGCACGATCTACGACATCGGTAGCAGCACGAACGTTCTGTACGCGGCCGCGGGTGGT
TCGGACGACTGGGCGAAGGGCGAGGGTAACGTGAAGTATTCCTATACCGTGGAATTG
AGAGATACTGGTAATTACGGCTTTGTTTTGCCTCCGGATCAAATCATTCCGACCGGT
GAAGAAACCTTCGAGGCGCTGAAGGTGGTGGCGAATTTCGTGAAGGACAACTATCGT
ACCCCGTAA

>Lv-CPB gene with introns (exons marked in dark background)
AGGCACCTTATATAGGCGGTTGCCAAGGCCGTGTTCTGCACAGACTCGCTCGCCATG
AGGTTCCTGGTCGTTCTCGCCTGCCTGGTGCTGGCGGCGCTCGCTCGCCCCAGCACG
CACGACTACCTGCACGGGTAAGAGGCTCGTTCCCCGGGGGTTGGGGCTGCTCGGGGG
GGTTGCGGCTGCTCGGGGGGGGGGCTGCTCGGGGGGGCTGGGGCTGCTCGGGGGGGT
TGGGGCTGCTCGGGGGGGTTGGGGCTGCTCGGGGGGGCTGGGGCTGCTCGGGGAGGT
CGGGGGGAGGGGGGAAGGCAGGGGGGGCGTTAGGGTCTGGCTTCTACGATGGTGGGC
TCTGCTTATAGCATGAGTGGGTTGCATAGCACACGAACTCACGAATACACTCACAAA
CGCGTTCATGTGCATTTATATATATAACATTATCATCATCATTATCCTGTTGCAGTC
CACTGCAGGAAGCCTCTCAATTTTTTTTTTTTTTTTATTGTTCCTGCCTTGCGTTT
TTCGTTTCCAGTCCTGGCCTAAATTTCGGATGTTTCGTCACGCCATCTTGTTACTGG
TCTTTTATTTCCTTTATATTTATTTCTTGTTATTTAAAGCCCAGCCTGTTACTTTC
TTCGTCCATCTGTCGTCTTGTCGCCTTCACATATGACCTGCCCTTCCCCATTTCTCC
TTTTCAATGCTCCTAATTATATCTTCCATGTTTGTCTGTTCTCTGATCCACGTCGCC
CTCATCCGATCTCTTAGGCTAATTCCCAGCATTGGTTAAAGACTTTTCTCTTTAGAC
GTAATAGCAGAGAACCTCCTAGTATGCTATTGTGTCTGAAAAGGGCGCTCCAGCCTA
GACTGATACATCGCTTTATTTTTATCTCTTCTGGATGTGTTTGTCTGTATAAACTGC
CATATATATATTTATGTTCGTATACTACATCAAGTACTTCGCCTTGTATATGTATCT
GTTCGAAGTGAACTCTATGATTGAACATGAGCTTAGTCTTTTTCCTGTTCATTTCGA
GTCCAACTTTCACTCTCTCTCTCTTCTCAGATCGTTTATCAACTTTCTCTCTCTCTC
TCTCTTCTCAGATCGTTTATCAACTTTCTCTCTCTCTCTCTTCTCAGATCGTTTATC

AATCACTCCAGTTCATCTGTTGCCAAAGAGAACAATATCGTCTCCAAATCTTAGGTT
GTTTAAAAGTTCGCACCGTACTTTCATGACCTTTCTAGTTAATGGAATAATTCTTTG
AGGCAAATTGTAAACAGTTTTGGCGAGATGGCGGCGTCTTGTCCAGCACCTTTTTAA
TGGGTGTTTTCTCTGTTTCTTTATGGAGCTTGATGGCTGCTGTCTCCTGTGCATCTG
TATCTTCCAATATTTTACAGTAGACTTCCTCTGTTCCTTGTCTTCGAATATATATAT
ATGTATATATATATATATATATATATATATATATATATATATATATATATATATATA
TGTATGTATGTATGTATGTATACACACACACACACACACACACACACACACACACAC
ACACACACACACACACACACACACACACACACACACACACACACACACACATATATA
TATATATATATATATATATATATATATATATATATATATGTATGTATATATATATAT
ATATATATATATATATATATATATATATATATATATATATATATATATATATATATA
TATATATATATATGTATATATATATATATATATATATATATATATATATATATATAT
ATATATATATATATTTATTTATCCACATATATATACATATATATATATATATATATA
TATATATATATATATATATATATATATATATATATATATATATATATATATATATAT
ATATATATATATATATATATATATATATACATATATATATATATATACATACATACA
TATATATATATATATATATATATATATATATATATATATATATATATATATGTATAT
ACATATATATATATATATATATATTTATATATTTATATATAGTTATATATATAGTTA
TATTTTTATATGTAGGTGTGTGTGTGTGTGTGTGTGTGTATGTATGTATGCATATAA
AGAGAGAGAGAGAGATAAAGAGATAGATGTAGATAGATAGTTATACATATATTTACA
GACACACACACACACAGATAGAGAGAGAGAAATAGAAAGAGAGAGAGAGAATAACAG
CCAGAAAAAAAACAGACATACATACAAACAGACAGAAGAAACAGACCAACACACTAA
AAAACAAAAAAACAAACAAGTCCAGCAATGAAAATAAATACCATAGGTCGCTCACAA
GATTTTTCTTTTTTCTTTTTCACCAGAGCTCAGGTTCTCCGAGTGAACCCCCAAACA
GCTGACCAGGTTCACTACCTCCAGGGTCTTCTGAAGACTGACCTGTATGACTTCTGG
ACTGAACCCCATGGCACTGGTACGTTTAAGTGATCTAGAGCCGGTTTGTTAAGTTTC
GCCTATCCGGACATGCGTGTAAAATTTAATGCATATCTGTGTGTCTGATAAATATGT
ATTTGTCTTCTATCTTCTATCCGGTTGATATACATGTCTAGACTGATAGATAGACAT
TTATCTATCTGCAGTCTGGTAGATATGCATCTCGACTGATATATGTGTTTTATACAT
ATTACTTATTCATAATCGACTTTGTTACTATTTCTATTCATCTATTCTTTTTTTTCT
TTTATAATTCTATTTCTCTATTTCTTTTTTCTATCTTTTTTAAAAGTCTATTTCTCT
ATTCCTTTTTTCTGTTTTAAAAGTCTATATCTCTATTTATTCATATTTTCTTTCTTT
TTTAAAAGTTCTTTTTTTAACAGTCTATTTCTCTATTCATTTTTTCTTTCTTTTTTA
AAGCTTATTTCTCTATTAATTTCTATCTTTCCTTTTTAAAAGTCTATTTCTCTATTT
TTTCTTTCGTTTTTAAAAGCATTTCCCTATTCATTTTCTTTCTTTCTTTAAAAAAAA
ATCTATTTCTCTATTAATTTTTCTTTTCTTTCTTTCCTTTTTATAAGCCTATTTCTC
TATTTTTTCTTTCCTTTTTAAAAATATTCCCCTATCCATTTTCTTTCTTCCTTTTAA
AAAAAATCTATCTCTCTACCAATCTTTCTTTTCTTTTCTCTCCTTTTCTCACAGGTC
ACCCAGTCGACATCATGGCCCAGGCATTTAGCGTCCCCGTCTTGAAGAAGACCCTCG
AGCAGATTGACCTCGACTTCACCATCCAGGTCAGCGACGTGGCCCTTCTACTGGCCA
AGGACAGGGAGGCCAACCAGAAGGCTCGAGCTGCTTCCGGGAAGGCCATGGACTGGA
CCTCGTACCATCGCTATGATGAGGTAGGGATAGGAGACTGAGTAGGGTATAAGGATA
GGAGACTGAATAGGGTATAAGGATAGGAGACTGAATAGGGTATAAGGATAGGAGACT
GAATAGGGTATAAGGATAGGAGACTGAGTAGGGTATAAGGATAGGAGACTGAATAGG
GTATAAGGATAGGAGACTGAGTAGGGTATAAGGATAGGAGACTGAATAGGGTATAAG
GATAGGAGACTGAGTAGGGTATAAGGATAGGAGACTGAGTAGGGTATAAGGATAGAA

GACTTAATTGGATATAAGGATAGAGAAATTGGGTACAAAGAGAGAGAACTAGATAGG
GTATAAGGATAGGAGACTGAATAGGGTATAAGGATAGAAGACTGAGTAGGGTATAAG
GATAGGAGACTGAATAGGGTATAAGGATAGAAGACTAAGTAAGGTATAAGGATAGGA
GACTGAATAGGGTATAAGGATAGAGAACTAAATCGGGTATAAGGATAGAGAACTCAA
TAGGATATAAGGATAGAGAAATTGGGTACAAAGATAGAGAACTAGATAGGGTATAAG
AATAGAAGACTAAGTAGGGTATAAGGATAGAGAACTAAATAGGGTATAAGGATAGAG
GACTTAATAGGTATAAGGATAGAAGACTGAATAGGGTATAAGGATAGAGAACTAAAT
AGGAGATAAGGATAGAGAAATAGGTATAAGGATAGAGAACTAAATAGGGTATAAGGA
TAGAGAAATAGGGTATAAGGATAGAAGACTAAAAAGGGTATAAGGATAGAGAAATAG
GGTATAAGGATAGAAGACTAAATAGGGTATAAGGATAGAGAACTAAATAGGGTATAA
GGATAGAAGACTAAATAGGGTATAAGGATAGAGAACTAAATAGGGTATAAGGATAGA
AGACTAAATAGGGTATAAGGATAGAGAACTAAATAGGGTATAAAGATAGAAGACTAA
AAAGGGTATAAGGATAGAGAAATTTGGTATAAGGATAGAAGACTAAATAGGGTATAA
GGATAGAGAAATAAGGTATAAGGATAGAAGACTAAATGGGGTATAAGGATAGAAGAC
TAAACAGGGTATAAGGATAGAGAATTAAATCGGGTATAAGGATAGAGAACTAAATAG
GGTATAAGGATAGAAGACTAAATAGGGTATAAGGATAGAGAAATAGGGTATAAGGAT
AGAAGACTAAAAAGGGTATAAGGATAGAGAACTAAATAGGATATAAGGATAGGAGAC
TAAATAGAAATATAAGGATAGAAGACTAAATAGGGTATAAGGATAGAGAACTAAATA
GGGCATAAGGATAGAAGACTAAATAGGATACTCAAACGCGCTGCCAATCCTTAACCC
AAAACCAAAACTCCCTCCCCCCTCCCCCTCCCTACCCCCACCCACCCCCTCACCCCT
ACCCCTCCCCTTCCCTCACCCCCCACCCACCTCCCCTTCCCTCACCCCCCACCCACC
TCCCCTTCCCTCACCCCTACCCCCCACCCACCCCCTCACCCCTACCACCCTCCCTCA
CCCCTCCCCTTCCCTCACCCCTCCCCTTCCCTCACCCCTCCCTCCCTCCCTCACCCC
TCCTTCCCTCACCCCTACCCCTCCCTCCCTCCCTCCCCCTTCCCTCCTCCCTCACCC
CTCCCCTTCCCTCCCCCTTCCCTCCTTCCCTCACCCCTCCCCTTCCCTCACCCTTCC
CTCACCCCTACCCCCCACCCACCTCCCCTCCCTACCCCCTACCCCCTCACCGCAGAT
CATGGCCTGGCTGGACGAACTGGCGTCCACGCACCCCAGTCTGTGCTCCGTGAAGGA
AGTGGGCACAACCTATGAGGGAAGGACCATGAAAATGCTGACGCTCAACAAGGGAGG
AACCGACAAGCCCGGGATCTTCATCGATGGAGGTGAGAGAAGGGAGAAGGAAGGAAG
GAGATAGGAGGAGAGAGAGAGAGAGTGAGGGGGGGGGGTAGAGAGAGAGGGAGGGAG
GAGGGAGGGAGAGAGAGAGAGGGAGAGGGAGGGGGAGAGAGGGGGGGAGGAGATAGG
AGGAGAAAGAGAGAGAGAAGGAGGGGGGGGGAGGAAGAGAGAGAGAGAAATAGAGAGTG
CGAAAGAGAGAGAGAGAAGGAGAGGGAGAGAGAGAGAGTGAGAGAGGGGGGGGGAGAGG
GAGGAAGAGAGTGAGTGAGAGAGAGAGATAGAGAGACTGAGAGAGAGAGAGAGAGAAAG
AGAGAGAAAGAGAGAGTAATGCGAAGAAGAGAGGAAATGGGATTGTATATTTTCAAA
AAAGAAAAAGAAGAATCTTTGATAAACATCTTGCAATTGGCTGAAATGTTTCTCTAT
GGTATTACTCATGTCACAGTGAAAAGTCATCTCAAAACATTATTGTTACTATCATTA
CGTTTCATTATTTCATTATTACTATTGACACCATTATTATTTTCATAATTGTCAACC
ATTATCATTATATGATTATCATTACTATTATTATCATCGTTGTTATTGGTATCATAT
CATCATTCCTGCCATCATCATCATCGTCATTATCATTATAACGATTATCACGATTGC
TGGTGCAGTATTATGATCCTTACAGTAATAATAAAATGGACACAATGTTATAATCAT
CATTAGCATCATCATTCTTATCGCAATCCCCACCATGACGAAACGAGTACAGAATCC
TCCCGTTTTTTTTTTCCCTTCGTCCTTCGTCCTTTTCATTTTCCTGTTCTTTCCCTT
CGTCTTTTTCATTTTCATTTTCATTTCTTTCCCTTCGTCCTTTTCATTTTCATTTTC

ATTTCTTTCCCTTCGTCCTTTTCATTTTCATTTTCCTTTCTTTCCCTTCGTCCTTTT
CATTTTCCTTTCTTTCCCTTCGTCCTTTTCATTTTCTTTTTCCTTTCTTTCCCTTCG
TCCTTTTCATTTTCATTTTCCTTTCTTTCCCTTCGTCCTTTTCCTTTTCCTTTCTTT
CCCTTCGTCCTTTTCATTTTCATTTTCCTGTTCTTTCCCTTCGTCCTTTTCATTTTC
ATTTTCCTTTCTTCCCCGCATCCGCCAGGCATCCACGCCCGCGAGTGGATTTCTCCC
GCCACCGTCACCTTTCCTTTCGTCCTTTCCTTTTCCTTTCTTTCCCTTCGTCCTTTT
CATTTTCATTTTCCATTCTTTCCCTTCGTCCTTTTCCTTTTCCTTTCTTTCCCTTCG
TCCTTTTCATTTACCTTTCTTCCCCTCATCCGCCAGGCATCCACGCCCGCGAGTGGA
TTTCTCCCGCCACCGTCACCTTTCCCTTCGTCCTTTTCCTTTTCCATTCTTTCCCTT
CGTCCTTTTCCTTTTCCTTTCTTTCCCTTCGTCCTTTTCATTTACCTTTCTTCCCCT
CATCCGCCAGGCATCCACGCCCGCGAGTGGATTTCTCCCGCCACCGTCACCTTTCCC
TTCGTCCTTTTCCTTTTCCATTCTTTCCCTTCGTCCTTTTCCTTTTCCTTTCTTTCC
CTTCGTCCTTTTCATTTACCTTTCTTCCCCCTCATCCGCCAGGCATCCACGCCCGCG
AGTGGATTTCTCCCGCCACCGTGACCTACATGCTGAACGAGCTGGTCACCAACAGCG
ACACCTACGACGACATCTTGTCCGCCGTGAACTTCTACGTTATGCCTTCCATTAACC
CCGACGGCTACGAGTACTGCCACACCGACGTAAGCTTAGGCCTGCCTGTTGTTTTTC
GGTGGGGGTGGGGAGGGGGAGAGGGGTGAAGGGGGAGGGGGAGGTCAGTAGCGACT
CGGGGAGGTGTCGTGTGTGGTGTCTGATTTTTTTTTTTTTTTTTTTTTTTTTTTTTT
TTTTTTTTTTTTTACGCGCGTGGGGTTTATTCTGATGACGTCACAGAAGTTGATAAT
AATAATGATTTTAATAATGATTTTAATTATCTTAATTAAGTAAAAATAATGATTTTA
ATGGTTATAATATTCATTGAACAATCATCAAAACAGACGCCATGACACCTCCTCCAG
TTGCTACTGATCTAACCTTCCCCTTGTTTATGGCGCGCTGTTTTATGGTGAAAATAA
TTAATAAAGATAGAACGAGATAAAAAGGTCAATAAATAGATGAGTAGACGATAAAAT
AGATGCATAGATATATGGATGAAAATGATTAATAAAAATACGAGGAGTATTTGACAA
ATTGATTCAAAACTAAACAAAAATCAGATTTTACGATAATGTTTTTATTCTTTAATT
TTGAATAGGTATTAATAAGTGTTATTTATGTATGAACACGTATATAAACGCACACAT
ACACACAGAAAAACCCATGTACACACATAAAAAAACATACACACCCAGACTCCAAAA
CACACACACAACCAGACACCAAACACAAAAATATATACACAAAAAAACACACATCCA
GTCTAAAAAATACACACACACCCAGACACCAAACACAAAAATAATCTCCATTTTCTC
CTTTTCTCTAAAGATAACAAAAAAAAATAAAAAAAATAAATAAATCTCTCTTCTTCC
TATCACACTCCCTTCCTAACGCACATCCTGACCCCAAACGCCAAAATAACAACCTCC
CTTTTCTCCCTTTCTATAAAGATAACAAAAATCAAGATAAATCATCTTTTCTCTCCT
ACAACAGGATAGGTTGTGGCGTAAGACTCGCTCTGACAACGGTTCTCCTTTCGGTTG
TAAGGGAGCCGATCCCAACAGGAACTGGGGACACCACTGGAACGGTTGGTACAATTT
GGGATTCTCATTTTCGTTTGGTATTATGTGTGGTATAATTGGTATTATTATTATTTA
ATATTCATTTTTATCATCTTTATTATTATGGTTGTAAGCTAGACTCGATAATTGGTA
TCATTGCTATTATTATTATTATTCGATATTCATTTTTATCGTTATTGTTATTATGGT
TGTTAAGCTAGACTTTCGGTAATGCGGTATTCACCAATATTATAACTTGGCCACTCA
CTAACACCATAAAAGCACCACCATTTCAACAGCTTTACTCGCCGTTCCATTCCAAAC
ACAACATTTTCACCATTCACCAACACCTTATGATTCACCAACACAACAAAGCCGATT
CCACTTCACTAGCACCATTATTTTACCATTCACCCACACCACTTTACCGTTCACCAC
CACAGCCTTACTCTTCACCACCATTACTTCACCATTCACCACCACAACATCATTCAT
CAACATAACTTCACCATTCACCAACACAAGTTCACCATTCGCTACAACTTCACCATC
CCCCATCACCCCTTCAACAACTTCTAACTCCCCCCTCCTCACTCTCACCCACCCCCT

CCTCCTCCCCCCTCCTCACTCTCACCCACCCCCTCCTCCTCCCCCCTCCTCACTCTC
ACCCACCCCCTCCTCACTCTCACCCACCCCCTCCTCCTCCCCCCCAGATAACGGCGC
CTCAGACAACCCCTCCTCACTCTCACCCACCCCCTCCCTCCTCCTCCCCCCTCCTCA
CTCTCACCCACCCCCTCCTCACTCTCACCCACCCCCTCCTCCTCACTCTCACCCACC
CCCTCCTCCTCCCCCCCAGAGAACGGCGCCTCAGACAACCCCTGCTCGGACATCTAC
GCCGGTCCCGAAGCCTTCTCAGAGATCGAGATGAAGAACGTGCGCAACCAGATCCTC
GACCAGACGAACCTGGTGGTGTACCTGACCTTCCACTCCTACTCCCAGCTGTGGCTC
TACCCTTGGGGCTACACTTCAGCTCTCCCCGAGGATTGGCAGGATTTGGTGAGTGTA
GCCGTGGGATTGGACGAGGACGCGGTGGTCGGATGGTTCAAGGGATCAAAGACTGTT
CGGGTGATCTGAGTTCGAGGCTTCGAGTCCCGGCCGGCGCTTTGTTTCCCTGTGGAA
TTTCACCTCTATTGACTATAGATATAGGGGGACGTGAAAGGAATAAGTCCATCGTAT
GTTCCTACGTGTCGTAAAAAAAAAAAGGTGACCATGGAAGCTTCTACCCAACCCACGC
TGGGCCAAGGAAGAGTATGGCCCACCCTCTCTCCATGATGGTACACGTTTTAAACAA
TATTAAGGCGGGACCTGTTGAGTCAGCGAGTTTCAAAGGAATATTAGAAACAGAACG
ATGTAGGGAAAGGTCTAAGGAAGGCTTATGTCCAACAACAGATGTAAAAGAGGCTTG
AAAAAATAGAAAGGGGTTTCGTCTGCTTTTTTTTTCTCTCTCTCTCTTTTTTACACA
TTCTTTTTTCCTTTTACACTTGGTTTAATTTTGAGTTTAAGCCTGAGTCTAAAGTTA
GTTTCGACACCAGCGCTGAAGTTTTGACAGAGCTCTGAAGCGGGTTCTGACACTGGG
TCTGGAATTAGCCTTGAGACTGTATCTGACACTGCCTTGAATACTGACTTGACACGA
GCTAGGAAGATGAATGGAATCTTTGCATTGACGTTTGCTCTAAAGTTGGTTTTTGTC
TTGCTTTAGACACTGGCTTTAAAAATAGCTTCGATCTTGGGTTCGATACAGGCTTTC
CACCATTTTTCTCTTTCAATAGAGTGAGATAGAGATAGAGACGGAGAATGAGAGAGA
AAAAAAAGAGAGAAAGAAAGAGAAAAAGAGAAACCATGAAACTTTGAGACAGAGATA
GAGACGGAGAATGAGAGAGAGAAAAAAAAAAGACAGAGAGAAAAAGAAAGAAAGAGAA
AAAGAGAAACCATGAAACTTTGAGACAGAGATAGAGACGGAGAATGAGAGAGAAAAA
AAAGAGAGAAAGCAAGAGAAAAAGAGAGACCATGAAACTTTGACACAGAGATAGAGA
CGGAGAATGAGAAAGAGAACGAGAGAGAGAGAGAGAAAAAAAAAAGACAGAGAGAAAA
AGAAAGAAAGAGAAAAAGAGAGACCATGAAACGTTGAAACCCAAAGCAAACTGATTC
ATTAACCAGTATCGATTAACAGAAGCTAATCCTTCCCTTGCAGGATGACTTGGCCCA
ATCCGCCGTGAACGCTCTCACAGCTGTGCACGGAACCATCTACGACATCGGATCCTC
CACCAACGTTTTGTGTGAGTTAAGAGCCATAGAGCTTTTTATATCACTCTATGAACT
TTTTATATCACTCTATGAACTTTTTATATCACTCTATGAACTTTTTATATCACTATG
AACTTTTTATATCACTCTATGAACTTTTTATATCACTCTATGAACTTTTTATATCAC
TATGAACTTTTTATATCACTCTATGAACTTTTTATATCACTATGAACTTTTTATATC
ACTCTATGAACTTTTTATATCACTATGAACTTTTTATATCACTCTATGAACTTTTTA
TATCACTATGAACTTTTTATATCACTCTATGAACTTTTTATATCACTCTATGAACTT
TTTATATTACTCTATGAACTTTTTATATCACTCTATGAACTTTTTATATCACTCTAT
GAACTTTTTATATCACTCTATGAACTTTTTATATCACTCTATGAACTTTTTATATCA
CTCTATGAACTTTTTATATCACTCTATGAACTTTTTATATCACTCTATGAACTTTTT
ATATTACTCTATGAACTTTTTATATCACTCTATGAACTTTTTATATCACTCTATGAA
CTTTTTATCACTCTATGAACTTTTTATATCACTCTATGAACTTTTTATATCACTCTA
TGAACTTTTTATATCACTATGAACTCTTTATGTCACTCTATGAACTTTTTATATCAC
TCTATGAACTTTTTATATCACTATGAACTTTTTATATCACTCTATGAACTTTTTATA
TTACTATATGAACTTTTTATATCACTCTATGAACTTTTTATATCACTCTATGAACTT

TTTTATATCACTCTATGAACTTTTTATGTCACTCTATGAAGTTTTTATATCACTCTA
TGAACTTTTTATGTCACTCTATGAAGTTTATATCCCTTAATGAACTTTCTATCAATT
TATGAGTGTTAAATATCAGTTTATGAACTTTCTATAGCAGTTTATAAGCTTTATATA
TCCCTCTATGAACTTCATATCACTTTATGAACTTTTTATGTCCCTTTATGAACTTTA
AATACCCTTTATGAACTATATACCCCTTTATGAACCTAATACACCTCCTTATGATCT
TTACCTATCCCTTTATGAACATTAAATATCCCTTCATGAACTATATATCCCTTTATC
AACTTTATACACTTCATTTTGGTCTTTATAGATCCCTTTATCAACCTTACACACTTC
ATTAAGGTTTTTATATACCCTTTTATGAACCTTAGATATACCATAATGAACTTTACA
TATCCCTTTATGAACCTTAGGTGCACCATTATGAACTTTACGTATCCCTTTATGAAC
CTTAAATATTATGAACTTTACATATCCCTTTATGAACCTTAAATATTATTAACTTTA
TATACCTCTTTATGAACCTTAAATACACCATTATGAACTTTACATACCCCTCTATGA
ACCTTAAATATCATGCACTTTACATACCCCTTTATGAACCTTAAATACACCATTATG
AACTTTACATACCCCCTTATGAACCTTAAATATACCATTATGAACTTTACGTATCCC
TTTATGAACCTTAAATATCATGCACTTTATGTATCCCCGAAGAACTTCCTCCCTCAC
CCTCTCCTCCCAACCCTCAGACGCCGCTGCCGGAGGGTCGGACGACTGGGCCAAGGG
CGAAGGCAACGTCAAGTACTCCTACACGGTGGAACTCCGAGACACGGGCAACTACGG
CTTCGTCCTTCCCCCTGACCAGATTATCCCCACCGGAGAGGAGACCTTCGAAGCCCT
CAAGGTCGTCGCGAACTTCGTCAAGGACAACTACCGCACGCCTTAGAGGGGGTGGTC
TCGCGAGAGGCTTGGACCGGACGTCTTGAAAGAAGAACGGCGAGGGTGTGTGGGTGG
GAGGAGGAAATAAATGTGATTTGTGGA
