# Supplementary material for: Molecular Characterization, Recombinant Expression, and Functional Analysis of Carboxypeptidase B in Litopenaeus vannamei
Source: Genes (Basel). 2025 Jan 9;16(1):69. doi: 10.3390/genes16010069 (PMC11764914; doi:10.3390/genes16010069)
Supplement: Supplementary file 1 [file genes-16-00069-s001.zip › Table S1 Primer sequences.pdf]

Table S1. Primer sequences information

Primers	Primer sequences (5'-3')	Amplicon length(bp)	Tm(℃)	% of efficiency
For cDNA cloning:				
Cpb-F1	CATCGGATCCTCCACCAACGTTT	303	66.9	88
Cpb-R1	TATTTCCTCCTCCCACCCACACA		65.2	
Cpb-F2	AGGCATTTAGCGTCCCCGTCTTG	297	68.5	84
Cpb-R2	TGTCGGTTCCTCCCTTGTTGAGC		67.3	
Cpb-F3	TTTCTCCCGCCACCGTCACCTAC	435	69.1	83
Cpb-R3	CATCCAAATCCTGCCAATCCTCG		67.5	
Cpb-F4	AGGCACCTTATATAGGCGGTTGCCA	1241	71.2	76
Cpb-R4	TCCACAAATCACATTTATTTCCTCC		68.1	
For qPCR				
q-Cpb-F	GCGTAATACGACTCACTATAGGGCG	274	61.5	81
q-Cpb-R	CATGATCTCATCATAGCGATGGTAC		60.6	
qβ-actin-F	CCGGCCGCGACCTCACAGACT	385	70.1	85
qβ-actin-R	CCTCGGGGCAGCGGAACCTC		69.7	
For ISH probe				
p-Cpb-F	ATGAGGTTCCTGGTCGTTCTCGC	636	62.6	82
p-Cpb-R	CGTCGTAGGTGTCGCTGTTGGTG		70.8	
For codon optimization:				
Cpb-CO-F1	TAGTGGTGGCGGTGGTTCAATGCAT CATCATCATCACCATAGTACCCATG ATTATCTGC	1271		
Cpb-CO-F2	GATCTCAATGATGATGGTGGTGATG CGGGGTACGATAATTATCTTTCACA AAATTGGCC			
