# Supplementary material for: Molecular Characterization, Recombinant Expression, and Functional Analysis of Carboxypeptidase B in Litopenaeus vannamei
Source: Genes (Basel). 2025 Jan 9;16(1):69. doi: 10.3390/genes16010069 (PMC11764914; doi:10.3390/genes16010069)
Supplement: Supplementary file 1 [file genes-16-00069-s001.zip › Table S2 Lv-CPB protein model quality.pdf]

Table S2. Lv-CPB protein model quality

Profile Type	Value
Template	A0A323TS92.1.A
Method	AlphaFold DB model
Oligo state	monomer
GMQE	0.93
Mol probity score	0.97
Ramachandran favored	96.89%
Bad bonds	0 / 3401
