# Supplementary material for: Molecular Characterization, Recombinant Expression, and Functional Analysis of Carboxypeptidase B in Litopenaeus vannamei
Source: Genes (Basel). 2025 Jan 9;16(1):69. doi: 10.3390/genes16010069 (PMC11764914; doi:10.3390/genes16010069)
Supplement: Supplementary file 1 [file genes-16-00069-s001.zip › Table S4 Comparison of CPB proteins between humans and shrimp.pdf]

Table S4. Comparison of CPB proteins between humans and shrimp

Aspect	Mammals	Shrimp (Lv-CPB)
**Tissue Expression**	Pancreas	Hepatopancreas
**Primary function**	Protein digestion; regulatory roles in inflammation and peptide processing	Protein digestion
**Optimal pH**	~7.5 (neutral)	~8.0 (slightly alkaline)
**Optimal temperature**	~37°C (body temperature)	Peak at ~50°C (wide temperature range)
**Zn^{2+} binding role**	Essential for catalytic activity	Essential for catalytic activity
**Additional roles**	Inflammation regulation, coagulation, peptide maturation	No evidence for broader physiological roles
